# Sleep: The Tip of the Iceberg in the Bidirectional Link Between Alzheimer's Disease and Epilepsy

**DOI:** 10.3389/fneur.2022.836292

**Published:** 2022-04-11

**Authors:** Anna B. Szabo, Benjamin Cretin, Fleur Gérard, Jonathan Curot, Emmanuel J. Barbeau, Jérémie Pariente, Lionel Dahan, Luc Valton

**Affiliations:** ^1^Centre de Recherches sur la Cognition Animale, Centre de Biologie Intégrative, Université de Toulouse, CNRS, UPS, Toulouse, France; ^2^Centre de Recherche Cerveau & Cognition (CerCo), UMR 5549, CNRS-UPS, Toulouse, France; ^3^Clinical Neuropsychology Unit, Neurology Department, CM2R (Memory Resource and Research Centre), University Hospital of Strasbourg, Strasbourg, France; ^4^CNRS, ICube Laboratory, UMR 7357 and FMTS (Fédération de Médecine Translationnelle de Strasbourg), Team IMIS, University of Strasbourg, Strasbourg, France; ^5^CMRR d'Alsace, Service de Neurologie des Hôpitaux Universitaires de Strasbourg, Pôle Tête et Cou, Strasbourg, France; ^6^Neurology Department, Hôpital Purpan Centre Hospitalier Universitaire de Toulouse, Toulouse, France; ^7^Toulouse NeuroImaging Center (ToNIC), INSERM-University of Toulouse Paul Sabatier, Toulouse, France

**Keywords:** Alzheimer's disease, epilepsy, memory consolidation, sleep, neuronal hyperexcitability, glymphatic clearance, interictal spike, EEG

## Abstract

The observation that a pathophysiological link might exist between Alzheimer's disease (AD) and epilepsy dates back to the identification of the first cases of the pathology itself and is now strongly supported by an ever-increasing mountain of literature. An overwhelming majority of data suggests not only a higher prevalence of epilepsy in Alzheimer's disease compared to healthy aging, but also that AD patients with a comorbid epileptic syndrome, even subclinical, have a steeper cognitive decline. Moreover, clinical and preclinical investigations have revealed a marked sleep-related increase in the frequency of epileptic activities. This characteristic might provide clues to the pathophysiological pathways underlying this comorbidity. Furthermore, the preferential sleep-related occurrence of epileptic events opens up the possibility that they might hasten cognitive decline by interfering with the delicately orchestrated synchrony of oscillatory activities implicated in sleep-related memory consolidation. Therefore, we scrutinized the literature for mechanisms that might promote sleep-related epileptic activity in AD and, possibly dementia onset in epilepsy, and we also aimed to determine to what degree and through which processes such events might alter the progression of AD. Finally, we discuss the implications for patient care and try to identify a common basis for methodological considerations for future research and clinical practice.

## A Brief History of Alzheimer's Disease and Epilepsy

In 1911, five years after presenting the first patient with the pathology that would later be named after him, Alois Alzheimer described a second case, a patient known in medical history as Johann F. Besides the progressive loss of cognitive functions and autonomy, the 56-year-old man experienced several epileptic seizures toward the later stages of the pathology (1, 2). This case, together with the detection of amyloid plaque-like structures called sclerotic plaques of neuroglia during the autopsy of an elderly epileptic patient in 1892, provided the first clues for a link between AD and epilepsy (3) [cited by Cipriani et al. (4)].

During the following century, the potential pathophysiological pathways of AD were meticulously investigated in a race to discover a treatment as the number of cases exploded, reaching ~33–38 million patients worldwide. However, the investigation of the AD-epilepsy axis failed to become what we would call today a “hot topic” in research. Sparse publications appeared every now and then, mainly advocating a link between familial forms of AD (FAD) and epilepsy. Patients with these rare forms, which account for ~1% of all AD cases and are due to mutations on the APP, PSEN1 or PSEN2 genes, were shown to have a high seizure incidence (5–11). As for sporadic AD, for quite a while, this comorbidity was defined as a mere marker of the severe stages of the disease (12, 13). A turn in these trends started with the seminal paper by Amatniek and colleagues in 2006 (14) which described an increase in seizure incidence among sporadic AD patients as of the earliest stages of the disease and which considered it a potential *part of the natural history of AD* (14). A smaller longitudinal study published the same year by Lozsadi and Larner echoed these results and suggested the existence of potentially shared pathogenetic processes between AD and epilepsy (15). Concomitantly, a series of studies in mouse models of AD (harboring mutations on the genes implicated in FAD) demonstrated the role of the AD-related protein-aggregates, namely Aβ plaques and neurofibrillary tangles [NFTs, (16)], in the observed neuronal hyperexcitability and spontaneous epileptic or epileptiform activity (17–20). (Note that for the remainder of this review, we will use Epileptic Activity/EA to refer to epileptic/epileptiform events). This brought about the introduction of the network dysfunction perspective in AD (21) and an explosion of publications on the link between AD and epilepsy in preclinical and clinical research.

The main objective of this review is to organize the available information fragments from these publications on the potential underlying causes and clinical consequences of the AD-epilepsy comorbidity and its apparent link to the sleep-wake cycle. Our secondary aim is to find ways to identify the pieces of the puzzle of the AD-epilepsy connection that are still missing by adapting current clinical practice and research methods. Therefore, we will first scrutinize the literature on pathophysiological processes to see how significant alterations in AD might trigger epileptic events. We will then attempt to distill coherence from the seemingly discordant clinical results on the characteristics of epileptic activity in AD patients. In the third and fourth parts, we will attempt to understand the possible consequences of these aberrant brain activities that occur during sleep on the progression of cognitive deficits in AD. Finally, we will examine how these pieces of information could be integrated into current clinical practice and research methodology that could increase coherence and comparability across studies.

## Potential Mechanistic Underpinnings of Ad-Related Neuronal Hyperexcitability

**Figure 1:**
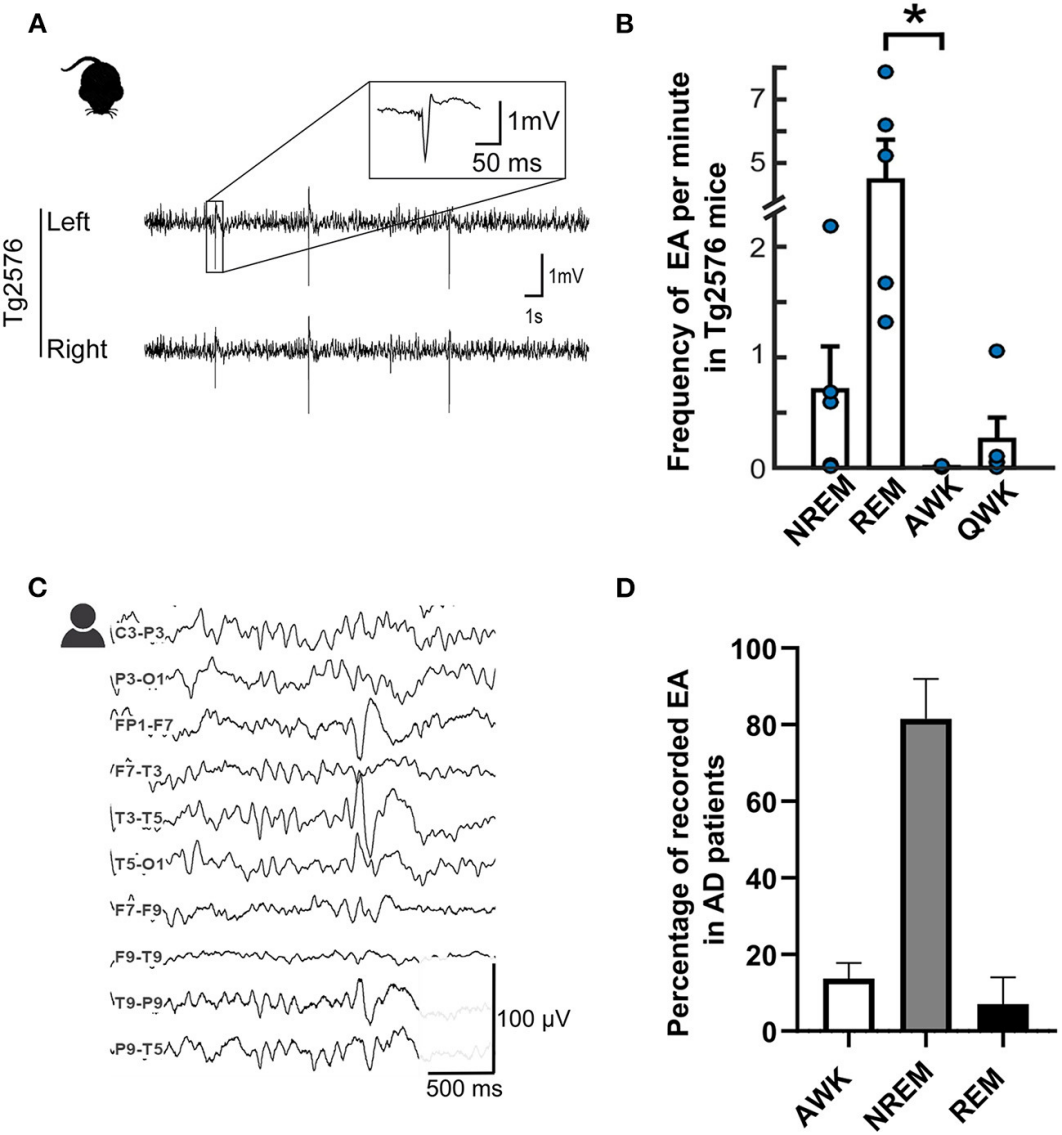
Epileptiform spikes in AD patients and the Tg2576 mouse model. **(A)** Representative examples of EA in Tg2576 mice (Power 1401 mk-II, CED, Cambridge, UK). Reproduced from (22) with permission **(B)** Distribution of epileptiform events in Tg2576 mice over the sleep-wake cycle in 6-week-old (*n* = 10, blue) and 6-month-old (*n* = 7, red) animals. Reproduced and adjusted from (23) with permission. **(C)** Longitudinal view of an epileptiform discharge during a vEEG examination, recorded from an AD patient at the University Hospital of Toulouse (Natus, Pleasanton, CA, USA). Calibration bar: 100 μV, 500ms. **(D)** Distribution of epileptiform discharges in AD patients in the studies reporting IED prevalence with awake and sleep-related data (24–26). For (24), data is pooled from AD patients with and without known epilepsy. Note that REM frequency was not reported for (25) and NREM stages were pooled together to account for differences in reporting methods.

Research over the past decade has made it abundantly clear that almost all mouse models used in AD research present aberrant network hypersynchrony and hyperexcitability quite early during the disease. These anomalous activities manifest as EA mostly during sleep or periods of inactivity in several models (Figures 1A,B), an aspect that, as we will see, seems to be similar in patients (Figures 1C,D). [For a more exhaustive list, see Kazim et al. (27)]. With the help of these models, significant advances have been made in elucidating the mechanistic pathways related to the aberrant network hyperactivity which have also been extensively reviewed recently (27–34). This review will only focus on the major pathways and the pathophysiological mechanisms related to clinical findings on the subject.

### Amyloid-β Accumulation and Neuronal Hyperexcitability

AD is well known for the diffuse presence of senile plaques in the brain. These deposits are composed of Aβ peptide aggregates resulting from the activation of the amyloidogenic proteolytic cleavage process of the APP protein by the β1 (BACE-1) and γ-secretases which leads to a particularly aggregation-prone form, Aβ_1−42_ [see (35) for an illustration]. Under physiological conditions, this pathway is only secondary to the non-amyloidogenic pathway which depends on cleavage by the α- and γ-secretases and leads to less aggregation-prone forms of Aβ. However, there seems to be slanting toward the BACE-1-dependent pathways in AD, resulting in the well-known extracellular aggregates first described by Alois Alzheimer (36), and translated by Stelzmann and colleagues in 1995 (37). Throughout the literature, the excitatory impact of Aβ as one of the main stimuli of neural hyperexcitability in AD is a surprisingly coherent finding. At non-pathological Aβ levels, Aβ production and neuronal hyperactivity seem to be part of a self-regulating feedback loop (38). However, in AD, this loop becomes a vicious cycle. In fact, increasing the Aβ load by blocking its degradation leads to over-excitation in the hippocampus (39), while elevated or decreased neuronal activity leads to increased or decreased Aβ aggregates, respectively (40–43). These findings are further supported by Busche and colleagues (17, 20). They showed that (I) hyperactive neurons cluster around Amyloid plaque and that (II) in the absence of plaque during the earliest stages of AD, even soluble forms of Aβ can drive neuronal hyperactivity in the hippocampus of APP/PS1 mice. The results obtained by Reyes-Marin and Nuñez (44) point in the same direction revealing a strong correlation between plaque load and epileptic events in the same model. Finally, Aβ clearance seems to decrease neuronal hyperexcitability (45), although some results are contradictory on this subject (46).

Various candidate pathways through which Aβ drives neuronal hyperexcitability have been suggested, including differential impacts of Aβ peptides on neural activity regulation depending on their state of oligomerization [(47), see (48) for a review on further hypotheses]). This could result in a steep Aβ peptide accumulation curve and, later on, amyloid plaques. Accumulating Aβ may then drive neuronal hyperexcitability through the dysregulation of calcium homeostasis due to a high plaque load (49, 50), which would be left unchecked due to the early deterioration of several types of interneurons.

### A Dysfunction of Fast-Spiking Interneurons

The above-mentioned dysfunctional interneurons are mainly parvalbumin-positive basket cells (PVBC) implicated in the synchronization of the activity of neuronal populations. Moreover, their dysfunction is strongly linked to cognitive impairment [for a summary of PVBC in AD, see Cattaud et al. (51)]. These interneurons seem to be dysfunctional in patients and hAPPJ20 mice due to decreased levels of the Nav1.1 subunit of voltage-gated sodium channels (52, 53). Interestingly, restoring the Nav1.1 subunit rescues both aberrant network activities and the memory-impaired phenotype of the hAAPJ20 model (52). Moreover, in the Tg2576 model (overexpressing a double mutant form of human APP695), Cattaud and colleagues recently demonstrated an early disruption of the perineuronal nets (PNN) surrounding the PVBC (51). PNNs not only protect cells from oxidative stress but they also play an essential role in stabilizing existing synapses, and through that function, memories (54, 55). Interestingly, restoring PVBC in the APP/PS1 model abolishes neuronal hyperexcitability and even improves cognitive function (56), which indicates that the observed PPN/PVBC-related deficits contribute to neuronal hyperexcitability in AD. Other populations of interneurons have also been described as suffering massive damage relatively early in APP/PS1 mice. This seems to be attenuated by the transplantation of embryonic interneuron progenitors that suppress the hyperexcitable phenotype (57). Nonetheless, the interneuron dysfunction observed in AD models seems to add insult to injury as it removes the brakes that could stop the runaway train of Aβ-induced hyperexcitability.

### Tau in Its Various Forms

In addition, the second major pathophysiological hallmark of AD after Aβ deposits, the intracellular aggregations called neurofibrillary tangles (NFT), also assist and even contribute to the hypersynchronous phenotype (21). NFT aggregations, which are thought to appear downstream to Aβ aggregation, are made of deposits of the hyperphosphorylated version of the microtubule-associated protein Tau (pTau). This hyperphosphorylated form leads to a loss of protein function and an increased probability of aggregation described by Mokhtar et al. (58). While many aspects of the pathways through which pTau or its endogenous form exerts a neurotoxic effect is still under investigation, the disentangling of their role in the epileptic phenotype associated with AD has started. For example, the reduction of total tau levels relieves neuronal hyperexcitability in mouse models of AD (19, 59). Furthermore, a recent longitudinal study in AD patients found that a risk of seizure was associated with higher total CSF tau levels (60), but not with higher pTau levels alone. In AD mouse models, the effect of tau on epileptogenesis seems to be driven by (I) the tau-dependent depletion of Kv4.2 potassium channels on dendrites (61) and (II) the interaction between endogenous tau, Fyn and PSD95. This latter interaction seems to be involved in the excitotoxic effect of Aβ as it increases the number of post-synaptic glutamate receptors and renders neurons more receptive to excitatory inputs (62). However, phosphorylating tau near to the microtubule domain renders this interaction impossible, leading to NMDA receptor endocytosis, which induces short-term neuroprotective suppression of hyperexcitable networks but comes with a long-term potential to induce network hypoactivity (63). Furthermore, Tau-hyperphosphorylation seems to be accelerated in a hippocampus subfield-specific manner after *status epilepticus* and during epilepsy (64). At the same time, neural activity and epilepsy-related accelaration of tau pathology has recently been demonstrated (65, 66). These results are coherent with the observations of Mondragón-Rodrígez and colleagues (63), who suggest that tau phosphorylation in the early stages of AD might be a neuroprotective mechanism against Aβ-related hyperexcitability to suppress neuronal hyperexcitability. However, in light of the recent results provided by Busche et al. (67), the respective roles of soluble and aggregated forms of pTau and the different ways through which endogenous Tau can increase or decrease neuronal activity in the presence of high Aβ loads needs further investigation.

### Dysfunctional Sleep-Related Systems in AD

Furthermore, the accumulation of all the aggregates and the subsequent aggravation of the epileptic phenotype are linked to one of the earliest clinical signs of AD: declining sleep quality.

Firstly, the progression of AD parallels a more and more fragmented sleep with extended awake periods during the night, increasing sleep latency and shortened total sleep time. The duration of REM sleep decreases, probably due to early atrophy of the brain areas in charge of cholinergic and noradrenergic transmissions that are essential for REM sleep integrity (68, 69). These disturbed sleep patterns may also contribute to the deposition of both Aβ and pTau and aggravate network hyperexcitability. In fact, Aβ load, as measured in CSF samples, fluctuates diurnally and increases during wakefulness (70) and even after a single night of sleep deprivation (71). In response, plaque accumulation was shown to aggravate sleep fragmentation in a drosophila AD model, while enhancing sleep duration led to decreased deposits (72). Interestingly, the same authors described a net excitatory effect of sleep loss and Aβ deposition leading to neuronal hyperexcitability that was responsive to ASM treatment (Levetiracetam, LEV) and that coincidentally prolonged the animals' lifespan as well. The disruptive effect of Aβ on sleep integrity and related memory consolidation has already been described (73), and in light of previous evidence on the subject, a bidirectional link between Aβ deposition and sleep fragmentation was proposed by Ju et al. (74). This link is such that low sleep efficiency turned out to be a good predictor of Aβ deposition rates over several years even for rather long durations (mean follow-up of 3.7 years ± 2.4) in healthy elderly adults (75). Another sleep-related marker, the decrease in Non-REM (NREM) slow-wave activity, was linked to Aβ deposition (73), and a similar or even stronger relationship was observed for tau deposits (76). More importantly, tau also seems to follow a sleep-wake cycle-dependent accumulation similar to Aβ (77).

**Figure 2:**
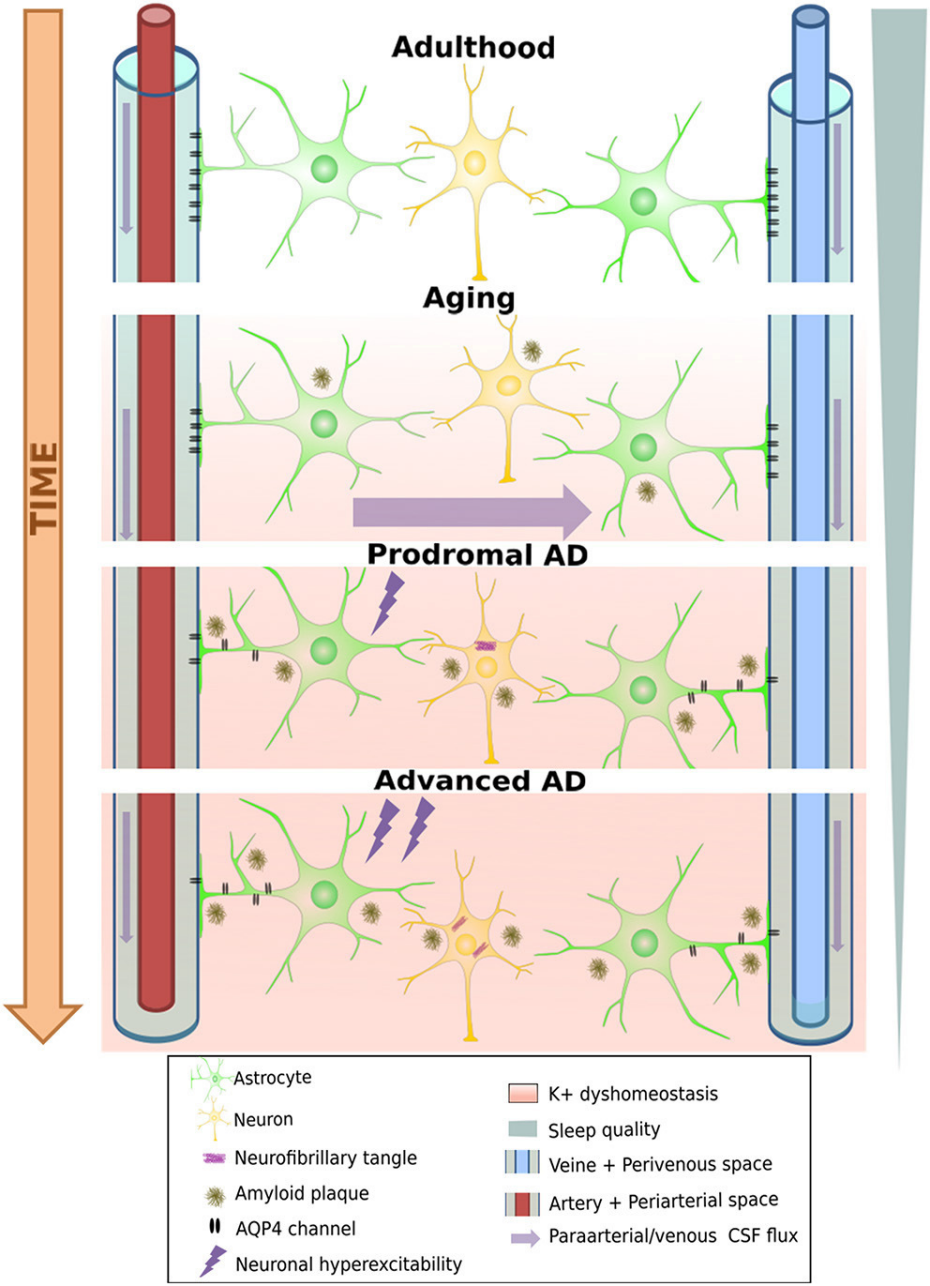
Degeneration of the glymphatic clearance system during healthy aging and Alzheimer's disease. White band represents time passing over the course of aging turning into neural pathology, including loss of AQP4 channels and their eventual loss of polarity on astrocyte endfeet. This leads to a loss of K+ homeostasis, which can already induce neuronal hyperexcitability, increasing A-beta production, further aggravating the process. On the other hand, progressive sleep loss also decreases glymphatic function, which amplifies all the other pathological processes mentioned as well.

This daily fluctuation is strongly linked to one of the crucial mechanisms by which the brain can eliminate excess or potentially toxic metabolites, including soluble Aβ and Tau: the glymphatic system. This relatively newly discovered clearance pathway (78, 79) is a highly organized fluid transport mechanism that accommodates an influx of subarachnoid CSF into the brain interstitium via intracerebral arterial perivascular spaces. Due to anterograde flow toward the venous equivalent of perivascular and perineuronal spaces that ends in the meningeal lymphatic drainage system, this mechanism can eliminate unwanted metabolites [Figure 2, see Rasmussen et al. (80)]. As our understanding of this system grows, it is becoming clear that dysfunctions in this pathway can cause rapid deposition of both Aβ and tau (81). Correct functioning of this pathway is highly dependent on water transport through aquaporin-4 (AQP4) water channels at astrocyte endfeet that line both the arterial and venous end of the perivascular spaces. These channels ensure low-resistance flow between perivascular spaces and brain interstitium (78), and several pathways by which these channels become dysfunctional in AD were recently uncovered (82).

First, these channels show an age-dependent decline even in wild-type mice (83), leading to a decrease by 40% of intraparenchymally injected Aβ-clearance efficiency. This is consistent with the findings that ~40–80% of all proteins and soluble metabolites are cleared via this mechanism (78). Importantly, it would appear that the glymphatic system is 90% more active during sleep than during waking hours (79). Given the considerable disruption of sleep and decreased total sleep time from the earliest stages of AD, it is plausible that the decrease induced in glymphatic clearance may further aggravate Aβ and tau accumulation (84). Moreover, fragmented sleep might do more harm than just decrease the “ON-time” of glymphatic clearance: Using two-photon *in vivo* imaging in non-transgenic mice, Liu et al. (85) showed that sleep deprivation leads to a mislocalization of AQP-4 channels that might render glymphatic clearance impaired. It is fascinating to note that in a transgenic model of AD (Tg-ArcSwe), Yang et al. (86) demonstrated an Aβ deposition-dependent mislocalization of AQP-4 channels on perivascular astrocytes even before the discovery of the glymphatic system. Importantly, they also drew attention to an earlier paper by Eid and colleagues (87), who found a dysfunction in perivascular AQP-4 channels in resected hippocampal sections from epileptic patients undergoing surgery. At that time, having no information about the existence of the glymphatic system, they hypothesized that such impairments may not only disturb water homeostasis but could also reduce K+ clearance, a function in which these channels were already known to be implicated. This K+ clearance deficit is yet another pathway that further spirals the hyperexcitable phenotype of AD mouse models (Figure 2). Moreover, a reactive state can also induce loss of polarity in astrocytes, as has been shown to occur near amyloid-β plaque in a transgenic FAD model (APP/PS1) (88). Deleting AQP-4 channels from the same model led to aggravated Aβ deposition both in the cortex and the hippocampus and resulted in an increased spatial memory deficits (89). In another “cross-transgenic” FAD model devoid of AQP-4 channels (5xFAD/AQP4 KO mice), an increase of ~6-fold in EA frequency was observed compared to 5xFAD mice at 10 months of age (90). Many questions remain about the glymphatic system and its relationship with AD (84, 88). Nevertheless, the possibility that yet another vicious cycle that amplifies amyloid deposition and epileptic susceptibility might exist seems plausible. In fact, age-related functional impairment of the glymphatic system may lead to increased plaque deposition. Plaque or Aβ_1−42_ oligomers could, in turn, create deposits in the vicinity of perivascular astrocytes, leading to a reactive glial state or the mislocalization of AQP-4 channels on astrocytes, further diminishing the process of glymphatic clearance (86, 88). This could lead not only to further acceleration of plaque deposition but also to a loss of K+ homeostasis, increasing the risk of epileptic activities, which, once again, leads to an increase in Aβ (and tau) load (Figure 2). Finally, the respiratory cycle also modulates glymphatic clearance. Inspiration is notably a strong driving force for the process in humans by its modulation on hydrostatic pressure which facilitates CSF flow toward the interstitial spaces of the brain (91). Importantly, breath-holding suppresses this flow and potentially decreases clearance, a scenario that may happen quiet often in patients with sleep apnea, which is a frequent comorbidity in AD patients (92). This is supported by the finding that the severity of obstructive sleep apnea seems to contribute to the build-up of amyloid deposition even in healthy older adults (93, 94).

Finally, it is of note that the glymphatic system, just as many physiological processes including the sleep-wake cycle, has been suggested to be under circadian control (95). Circadian rhythms are maintained via a complex transcriptional machinery that are present in most cells in the human body and are modulated via the suprachiasmatic nucleus of the hypothalamus that is thought to serve as a “master body clock” (96). Interestingly, both clinical and preclinical research have suggested a dysfunction in the circadian rhythms in AD that might happen even before the onset of the clinical symptoms of the pathology. Albeit the mechanistic pathways of the circadian clocks are outside of the scope of this review, this angle needs further investigation as mending this dysfunction that is upstream of several pathways by which AD might progress and by which EA might appear could have therapeutic implications (96).

### A Potential Bidirectional Road to Accelerated Disease Progression

As mentioned earlier, many other mechanisms have been suggested, such as the APO-ε4 allele, (97–99), and an early reduction in the level of either the mitochondrial protein deacetylase sirtuin 3 (100) or reelin [(101, 102), reviewed by (103)].

All these converging and synergistic pathways introduced in this paragraph could contribute to forming a dysfunctional network in which the excitation/inhibition balance has been considerably slanted toward hyperexcitability, which makes the occurrence of epileptic events or even seizures highly probable. Neuronal hyperactivity during such events might in turn further aggravate the situation by reorganizing functional networks (18, 104, 105) and generating substantial amounts of Aβ. Since the important pathological pathways that lead to AD can be triggered decades before the onset of cognitive symptoms, these processes can slowly but steadily reorganize and destroy existing functional circuits. This also applies to sporadic forms of AD, where epilepsy might not be as clear-cut a symptom as in the case of FAD, yet silent reorganization might occur during the asymptomatic period of plaque and NFT accumulation. This could lead to aberrant network activities, even before cognitive symptoms appear (25).

The suggestion of a disease-accelerating synergistic phenomenon between AD and epilepsy also begs the question of the possible bidirectionality of the link between the two pathologies (48, 106). Clinical research has suggested that age-related senile plaque deposition is more significant among TLE patients than healthy older adults. Moreover, an enhancing effect of the epileptic syndrome on plaque formation and amyloidosis has been suggested. This is based on observations from resected cerebral tissue and CSF AD-biomarker measurements on epileptic patients, as well as observations from genetic and neurotoxic AD mouse models (107–109). A similar increase to that of amyloid burden was found for pTau in post-surgery brain resections collected from TLE patients with focal drug-resistant epilepsy (65). Moreover, a very recent review by Tombini et al. (34) showed that TLE and AD patients share many pathophysiological associations starting with the presence of hallmark depositions of AD (Aβ plaques and NFTs, or their soluble forms), a similarity that is more robust for late-onset TLE cases (110).

However, does the current clinical research confirm (I) the vicious cycle of Aβ, NFTs and EA promoting a fast accumulation of aggregates and a consequent fast progression of AD, and/or (II) the bidirectional relationship between AD and epilepsy?

## Filtering Coherence From Discordant Findings

First of all, it is essential to note that the prevalence of subclinical epileptic activities (Table 1) and epileptic seizures (Table 2) in AD patients is highly variable across studies, reaching 64% (133) in some cases while barely hitting 2% in others (124). This is probably due to the extreme variability in data collection methods (134), an issue we will discuss in detail in the last paragraph. However, several coherent findings emerge from the apparent discordance that can guide our understanding of this peculiar link between AD and epilepsy, and we will first focus on these points.

**Table 1:** Main inclusion criteria and results of clinical studies exploring the prevalence of epileptic activities in AD patients.

**References**	**Mean age in years ± SD (*n*/group)**	**AD diagnostic criteria**	AD severity (MMSE/ACE/CDR mean + SD^*^)	**History of epilepsy**	**Excluded medications or substances**	**EEG type and duration**	**EA detection**	**% with EA or incidence**	**Risk factors for EA**
Liedorp et al., (111)	**1. AD–no EA**: 63 ± 10 (**500**) **2. AD+EA**: 71 ± 9 (**10**)	NINCDS-ADRDA	**MMSE** = 25 ± 5	Included	NS, but accounted for in the analyses	Standard 30-min EEG	1 neurophysiologist	**2%** of AD cases	Younger age, Disease severity (trend)
Vossel et al., (112)	**At diagnosis:** **1. AD+EPI**: 69.1 ± 9 (**35)** **2. AD-no EA**: 74.5 ± 10.3 (**969**) **3. MCI+EPI**: 68 ± 7.8 (**12**) **4. MCI-no EA**: 74.6 ± 9.5 (**216**)	**1. AD:** NINCDS-ADRDA **2. MCI:** IWG	**MMSE range** **=** 4–30	Included	Alcohol/substance abuse	Standard 20-min EEG for a sub-group of 152 participants	Multidisciplinary team of experts	**Groups 1, 3:** 24/39 **Groups 2, 4:** 7/113	Younger age at onset
Vossel et al., (25)	**1. AD** 61.7 ± 7.4 for (**33**) **2. CTRLs**: 65.3 ± 5.6 (**19**)	NIA-AA	**MMSE range**: **1. AD** = 18–24 **2. CTRLs** = 29–30	Not included	BZD Antipsychotics Narcotics Antihistamines. Substance abuse	Overnight vEEG and 1h M-EEG	1 epileptologist + 1 neurophysiologist per exam type, blinded	**1. AD**: 42,4% **2. CTRLs:** 10.5%	No risk factor, but faster decline
Horvath et al., (26)	**1. AD-no epilepsy:** 79.4 ± 7.4 (**20**) **2. AD** **+** **seizures**: 75.9 ± 4.7 (**10**); **3. AD+IEDs:** 73.6 ± 9.3 (**12**)	NINCDS-ADRDA	**ACE** **1: AD-no EA** = 67.4 ± 9.4; **2. AD** **+** **seizures** **=** 38.4 ± 15.3; **3. AD** **+** **IEDs** **=** 44.5 ± 7.4	Included (if onset <10y before AD)	Antipsychotics Antidepressants Antihistamine BZD	24 h ambulatory EEG	Two independent neurophysiologists (1 blinded). ILAE definition of epilepsy diagnosis	28% IED without seizures + 24% with seizures	AD severity; High VLOM ratio; Higher educational level
Brunetti et al. (113)	**1. AD**: 73 ± 7 (**50**) **2. MCI:** 72 ± 6.7 (50) **3. CTRL:** 69 ± 6.7 (**50**)	NIA-AA and DSM-IV	**MMSE** **=** 18.38 ± 4.7	Not included	Psychoactive/hypnotic drugs History of alcohol/ substance abuse	Full-night vEEG (134/150 participants)	Automatic followed by two neurophysiologists' verification (blind)	**AD**: 6.38% (3/47) **MCI**: 11.63 (5/43) **CTL**: 4.54% (2/44)	-
Lam et al., (24)	**1. AD-no EA** 76.3 ± 7.2 (**41**) **2. AD** **+** **EA** 69.6 ± 7.6 (**15**) **3. CTRLs:** 72.6 ± 9.1 (**43**)	NIA-AA	**MMSE range**: 13–30	Included (separate group)	BZD/“sleep aids” AMS (for groups 1, 3)	24 h ambulatory EEG	**Two steps**: **1**. 2 epileptologists **2**. A panel of 9 epileptologists	**AD-no EPI:** 22% **AD+EPI**: 53.3% **CTRL**: 4.7%	SSS-like waveforms; Earlier age of onset
Vossel et al., (114)	**1. UCSF branch**: 60.2 ± 7.1 (**18**) **2. UMN branch**: 64.8 ± 7.8 (**16**)	NIA-AA	**MMSE** > 17 **CDR** <2	Included (if onset <5y before screening)	BZD ASM Narcotics	Overnight vEEG and 1 h M-EEG	1 epileptologist and 1 clinical neurophysiologist (both blinded); + Persyst EEG software	38,20%	Tendencies (p = 0.054) for females and APOE4 carriers toward AD + EA

* *Unless otherwise stated. ACE, Addenbrooke's Cognitive Examination; AD, Alzheimer's Disease; ASM, Antiseizure Medication; BZD, Benzodiazepines; CDR, Clinical Dementia Rating; CTRL, Controls; EA, Epileptic/Epileptiform activity; IED, Interictal Epileptiform Discharge; IWG criteria, AD diagnostic criteria based on the propositions of the International Working Group; MCI, Mild Cognitive Impairment; MMSE, Mini Mental State Examination (Folstein version); NIA-AA criteria, AD diagnostic criteria based on the propositions of the National Institute on Aging and the Alzheimer's Association; NINCDS-ADRDA criteria, AD diagnostic criteria based on the proposals of the National Institute of Neurological and Communicative Disorders and Stroke and the Alzheimer's Disease and Related Disorders Association; NA, Not applicable; NS, Not Specified; vEEG, Video EEG; VLOM Ratio, Ratio of Verbal Fluency and Language ACE subscores over the Orientation and delayed recall subscores*.

**Table 2:** Main inclusion criteria and results of clinical studies that explore the prevalence of seizures in AD patients.

**References**	Age mean in years ± SD^*^(*n*/group)	**AD diagnostic criteria**	**Disease severity** **(MMSE/ACE/CDR** mean + SD^*^)	**History of epilepsy**	**Excluded medications or substances**	**EEG type or seizure information source**	**Seizure detection**	**% with seizures per group or incidence**	**Risk factors for seizures/epilepsy diagnosis**
Romanelli et al., (12)	**1. AD**: 71.5 ± 4.9 (**44**) **2. CTRL:** 71.7 ± 4.9 (**58**)	Criteria from Morris et al., (115)	NA	Not included	- Alcohol abuse - “Other medical illnesses” with a higher risk of seizures	- Standard EEG - Questions at follow up - Medical/ Nursing home records	Questions at follow up; medical records; nursing home records	15.9% of patients, 0% of controls	- Severe stage
McAreavey et al., (116)	**Age** **>** 55 (208 dementia cases)	ICD-9	**MMSE range**: 0-24 **MMSE means**: **1. Seizure**: 2.5 **2. No seizure**: 4.8	Included	NS	- Questions to nursing and medical staff	- ASM prescriptions - Medical records - Nursing home staff	9.1% (84% of those with AD)	- Younger age - Earlier age at admission
Mendez et al., (13)	**Age NS** (**446** AD patients)	Medical records and autopsy	NS	Included	NS, but alcohol abuse and “other medical illnesses” accounted for	- Questions to family members, nursing home staff and physician	- Medical records - Nursing home staff - Physician - Questionnaire for family members	17.30%	- Younger age of onset, - Advanced AD stage
Volicer et al., (117)	**1. AD+EA**: 70.6 ± 4.4; **2. AD-no EA**: 72.4 ± 3.5 (**75** DAT altogether)	NINCDS/ADRDA or DSM IIIR	NS for all patients, but severe cases	Included	NS	- Interview with caregiver if history of seizures was found	- Observation - Medical records - Interview with caregiver	21% after being institutionalized (36% when pre-existing epilepsy counted)	- Faster decline, especially in language ability
Lozsadi and Larner, (15)	**Range at diagnosis**: 49–84 (**177**)	NINCDS-ADRDA	NS	Included	No	- Medical records	From medical records, classification based on ILEA criteria	6.8%	- NS
Amatniek et al., (14)	NS, but age > **50** (**233** probable AD)	NINCDS-ADRDA	**MMSE** ≥ 16	Not included	Antipsychotics Drug/alcohol abuse	- Standard EEG for 58.37% of participants - Question on seizure occurrence since last visit	Two neurologists' evaluation based on questionnaires and medical records	0.87%	- Younger age - African-American ethnicity. - AD severity at initial visit - Longer duration of symptoms - Lower educational level - Focal epileptiform findings - Depression (for younger participants)
Rao et al., (118)	**Mean: 76.9** in the seizure group (**1,738** dementia cases altogether)	Medical records	NS	Included	NS	- EEG for 74% of patients with seizures - Medical records	Medical records	3.6% (2.24% confirmed, 48.7% of those had AD/MCI)	-
Scarmeas et al., (119)	74.4 ± 8.9 at entry (**453** AD)	NINCDS-ADRDA or DSM IIIR	**Baseline MMSE**: 21 ± 3.3 All patients > 15	Included	None, but many taken into account in the analyses	- EEG for 21 out of 52 with suspected epilepsy - Follow-up interview every 6m - Medical records	2 epileptologists reviewed medical records and interviews	1.5% of patients	- Younger age
Bernardi et al., (120)	78 ± 7.2 (145 probable AD)	NIA-AA	**MMSE** = 19.9 ± 6.3 (Range: 3-27)	NS	None but: antidepressants + antipsychotics accounted for	- EEG for 21/145 patients at baseline (and for all with identified seizures) - Medical records	Based on ILAE criteria (63), by one epileptology expert	9.7% of AD	- Gender (male) - AD severity - Hyperlipemia
Irizarry et al., (121)	74.5 ± 9.5 (**3,087 AD** from 10 previous studies)	NINCDS-ADRDA	**MMSE range:** 10–28	Not included	No, but taken into account in the analyses	- Only data available from the previous studies	Verbatims from clinical trials	4.84/1,000 py	- Younger age - AD severity at baseline - Antipsychotic use
Imfeld et al., (122)	**1. AD**: 80.7 ± 6.7 (**7,086**) **2. CTRLs** **~age matched (7,086)**	Algorithm, from diagnostic coding from GPs	**NS**	Not included	No, but verified for antipsychotics + antidepressants	- UK General Practice Research Database	From GP coding	**AD**: 5.6/1,000 py; **CTRL**: 0.8/1,000 py	- Longer disease duration
Vossel et al., (112)	**At diagnosis: 1. AD+EA**: 69.1 ± 9 **(35)** **2. AD-no EA**: 74.5 ± 10.3 (**969**) **3. MCI+EA**: 68 ± 7.8 (**12**) **4. MCI-no EA**: 74.6 ± 9.5 (**216**)	**1. AD**: NINCDS/ADRDA **2. MCI**: IWG	**MMSE range:** 4–30	Included	Alcohol/substance abuse	- Standard 20-min EEG for a sub-group of 152 participants; - Medical History	Multidisciplinary team of experts	2.86% of AD, 5.26% of MCI	- Younger age at onset
Cook et al., (123)	**Mean** ≈ 80 (*n* = **11,042** AD and **11,042** non-AD dementia)	- Diagnosis codes CPRD	NS	NS	Antipsychotics + antidepressants accounted for	- Diagnosis codes from GPs	GP diagnosis codes and follow-up questionnaire	**AD**: 8.8/1,000 py **Non-AD dementia**: 1.7/1,000 py	- Stroke - Antipsychotics prescribed within 6 m
Giorgi et al., (124)	**At diagnosis**: 69.6 ± 8.5 (1223 AD)	NINCDS-ADRDA	**MMSE Range** = 3–29 **Median** = 18.67	Included	NS	- EEG for a subset of patients only - Medical records	From medical records	2.45% (1.63% without patients with concomitant lesions)	- NS
DiFrancesco et al., (125)	**At onset:** 75 ± 7 (1,371)	NIA-AA criteria	NS, probably advanced	Included	Antipsychotics Antidepressants Alcohol/drug abuse	- EEG for a subset of patients only - Medical records	From medical records (with EEG when available)	1.68% before AD + 1.16% after AD onset	- Earlier onset of cognitive decline
Horvath et al., (26)	**1. AD-no EA:** 79.4 ± 7.4 (**20**) **2. AD+seizure**: 75.9 ± 4.7 (**10**) **3. AD+IEDs:** 73.6 ± 9.3 (**12**)	NINCDS-ADRDA	**ACE** **1: AD-no EA** = 67.4 ± 9.4; **2. AD** **+** **seizures** **=** 38.4 ± 15.3; **3. AD** **+** **IEDs** **=** 44.5 ± 7.4	Included (if onset <10y pre- AD)	Antipsychotics Antidepressants Antihistamine BZD	−24h ambulatory EEG - Epilepsy-related information collection	2 neurophysiologists (1 blind to condition), ILAE definition of epilepsy diagnosis	24%	- AD severity - High VLOM ratio - Higher educational level
Rauramaa et al., (126)	85 ± 8.6 (**64** AD - 7 mixed dementia)	NINCDS-ADRDA and autopsy	NA	Included	NS (but no alcohol abuse)	- For 10/11 patients with epilepsy - Medical records	EEG, medical records	17.20%	- Younger age of onset - Longer disease duration
Baker et al., (127)	75.1 ± 7.07 (***n*** **=** 72 at 1-year follow-up)	NIA-AA	**CDR-score at BL** = 4.2 ± 2.69)	Included	NS	- Structured interview (with informant)	From interview	**At follow-up: Probable:** 18.06%; **Possible:** 19.44%	- Worse score on CBI-R at baseline - Worse cognitive functions at follow-up (problem solving, personal care, attentional capacities ↓, daytime sleeping, confusion, fluency difficulties ↑)
Lyou et al., (128)	**Age** **>** 70 (**4,516** AD; **19,713** CTRL)	ICD-10	NS	NS	No	- Medical database	Diagnostic codes from medical database	**At last follow-up** **1. AD:** 13.97 % **2. CTRL**: 6.05%	- Gender (male) - Hypertension or hyperlipidemia - Chronic kidney disease
Tabuas-Pereira et al., (60)	**1. AD+EA:** 68.4 ± 8.4 **2. AD-no EA**: 68.1 ± 9.9 (**292** AD altogether)	NIA-AA	**MMSE** **1. AD** **+** **EA** = 16.2 ± 64 **2. AD – no EA**: 20.8 ± 7.4	Not included	NS on medication No alcohol abuse	- EEG for patients with suspected epilepsy - Medical charts	Retrospectively from medical files, backed by EEG	17.8%	- Earlier onset of dementia - Lower MMSE even at baseline - Higher tau load
Stefanidou et al., (129)	**1. Dementia**: 83 ± 7 (**660**) **2. CTRLs**: 83 ± 7 (**1,980**)	DSM IV criteria	NA	Not included	NS	- EEG at least for a subset, but NS - Medical charts - ICD-9 codes - Follow-up interviews	Scoring of epilepsy probability by 2 epileptologists, based on ILAE criteria	**1. Dementia group**: 2.9% **2. CTRLs**: 2%	-
Vöglein et al., (130)	**1. AD pre-symptomatic.:** 79.8 ± 7.7 (498) **2. Impaired-no** **-MCI**: 74.9 ± 9.0 (43) **3. AD MCI:** 75.1 ± 8.3 (859) **4. AD**: 75.1 ± 9.9 (9,127) **5. CTRLs**: 69.8 ± 10.9 (10,218)	NINCDS/ADRDA or NIA-AA	**MMSE** **1. Pre-sympt**.: 28.2 ± 1.8 **2. Impaired-no MCI**: 27.2 ± 2.6 **3. MCI**: 26.1 ± 2.7 **4. AD**: 19.5 ± 6.7 **5. CTRL**: 28.9 ±1.4	Included	NS	- Interview with participant and “co-participant” - Medical records - Observation	- Interview with participant and “co-participant” - Medical records - Observation	−2.93% all confounded - 3.14% of AD	- Earlier age of AD onset - Worse cognitive and functional state - Longer AD duration
Zelano et al., (131)	**Age range** = 32–107 [(25,626 non-mixed dementia AD case); 223,933 CTRLs]	ICD-10	NS, probably broad range	Included	NS	SveDem database	ICD-10 and ICD-9 codes, meeting ILAE criteria	**1. All cases**: 4%, **2. LOAD**: 2.1% **3**. **EOAD**: 5%	- Young age, - Gender (male) - History of stroke/head trauma/brain tumor, - Lower MMSE
Blank et al., (132)	**Median** **=** 84 (178,593 probable AD	Medical records from various sources	NS	NS	NS	- Medical records	Mention of epilepsy diagnosis in records	4.45% of AD	- Stroke - Depression - African-American ethnicity

* *Unless otherwise stated. ACE, Addenbrooke's Cognitive Examination; AD, Alzheimer's Disease; ASM, Antiseizure Medication; BZD, Benzodiazepines; CDR, Clinical Dementia Rating; CPRD, Clinical Practice Research Datalink; CTRL, Controls; DSM-IIIR, Diagnostic and Statistical Manual of Mental Disorders – 3^rd^ revised edition; EA, Epileptic/Epileptiform activity; ICD-9/10, International Classification of Diseases; IED, Interictal Epileptiform Discharge; IWG criteria, AD diagnostic criteria based on the propositions of the International Working Group; MCI, Mild Cognitive Impairment; MMSE, Mini Mental State Examination (Folstein version); NIA-AA criteria, = AD diagnostic criteria based on the propositions of the National Institute on Aging and Alzheimer's Association; NINCDS-ADRDA criteria, AD diagnostic criteria based on the propositions of the National Institute of Neurological and Communicative Disorders and Stroke and the Alzheimer's Disease and Related Disorders Association; NA, Not applicable; NS, Not Specified; py, person-years; vEEG, Video EEG; VLOM Ratio, Ratio of Verbal Fluency and Language ACE subscores over the Orientation and delayed recall subscores*.

The first of these coherent findings is that AD-related EA is primarily subclinical and, therefore, hard to detect. This might explain why this comorbidity has been long overlooked. On one hand, these EA include subclinical epileptic spikes without a behavioral output and which are, as we shall see, difficult to detect by conventional non-invasive methods (135). On the other hand, focal seizures with temporary loss of contact in AD patients might be interpreted as a symptom of AD instead of silent epileptic seizures (26, 112). Therefore, there is a high chance that aberrant network activities go unnoticed and untreated for extended periods.

The second point of cross-study consensus is that the progression of AD in patients with a co-occurring epileptic syndrome or epileptiform activities is faster (25, 117, 130, 136). Moreover, AD patients with EA seem to have worse results on cognitive tests than patients without such aberrant network activities (26, 120, 121, 137). This correlation does not seem to be merely related to a longer duration of AD or more severe stages of the disease. First, Vossel et al. (25) demonstrated that even if patients with and without EA did not differ at baseline in their mini-mental state examination (MMSE) scores, a follow-up over several years showed a steeper cognitive decline for patients with EA. Second, Vöglein and colleagues (130) recently showed that even after adjusting for age and disease duration, the MMSE scores were still unexpectedly low for AD patients with underlying epileptic activity. Moreover, they found that a history of seizures had a significant negative impact on disease severity (as measured by the Clinical Dementia Rating Scale–Sum of Boxes/CDR-SB). Finally, a recent study of Horvath and colleagues described a 1.5 times faster decline of global cognitive performances of AD patients with EA compared to patients without epileptic abnormalities on their EEGs (136).

Another coherent finding is that AD patients with comorbid epileptic activities experience an earlier age of onset of cognitive symptoms (14, 24, 60, 112, 119, 121, 130, 138). This, as we will detail further below, is indicative of a synergistic accelerating of AD- and epilepsy-related factors on cognitive decline which could lead to a faster progression and an earlier age of onset instead of the decade-long process of insidious plaque and NFT deposition.

The localization of EA also seems to be a relatively coherent cross-study finding. Similarly to AD-independent mesial-temporal lobe epilepsies (mTLE), these aberrant network activities in AD are predominant in the temporal lobes and more often detected on the left hemisphere by EEG (24, 25). However, concerning the study of Lam and colleagues (24), it is of note that while left temporal subclinical EA were indeed highly predominant in AD patients with no history of epilepsy, patients with a history of seizures were more prone to have right and left temporal interictal discharges as well.

Last but not least, one of the cornerstones of this review is the strong relationship between the sleep-wake cycle and EA, since sleep-related increase in EA is one of the most consistent findings across both clinical and preclinical literature (24–26) (Figures 1C,D). This sleep-related increase in EA implies that they could plausibly interfere with sleep-related memory consolidation, one of the major potential consequences of AD-related epilepsy which we will examine in the next paragraph.

## The Impact of Subclinical Epileptic Activities on Memory Deficits in AD

An increase in EA during sleep in AD patients is not surprising as such a sleep-related increase in the occurrence of epileptic events has already been shown in non-AD epileptic patients (139). However, in epileptic patients, EA seems to fall during periods of NREM sleep and is extremely rare in REM sleep (139). In FAD mice (hAPPJ20 or Tg2576), this is not entirely the case. While EA is still predominant during inactive periods (52) such as sleep, their frequency shows a drastic increase during REM sleep compared to NREMS (23, 140), pinning down a visible discrepancy between animal and human results (140). This pattern is undoubtedly puzzling, as REM sleep is thought to be highly protective against EA due to the desynchronized EEG patterns that make the occurrence of hypersynchrony-related events rather unlikely (139).

It is of note that the predominance of EA during NREM sleep in clinical results might be partially due to difficulties in detecting REM sleep in AD patients, as notable EEG slowing specifically during REM sleep has been observed in AD, which might impede correct scoring of REM sleep (141, 142). However, currently available clinical data (albeit scarce for the moment, Figure 1C) are still more compatible with what is observed in non-AD epileptic patients, with EA predominantly occurring during NREM sleep (Table 2). Consequently, while we look at the potentially detrimental impact of EA on memory consolidation in the next paragraph, we mainly focus on NREM sleep and less on REM sleep.

### The Orchestra of Oscillations Behind Memory Consolidation

Throughout the past fifty years, an impressive panoply of theories have been proposed and refined concerning the role of sleep in memory consolidation and this was extensively reviewed (143–145). A theory of a memory consolidation mechanism was first postulated by Marr in the early seventies (146, 147). He suggested that the hippocampus might only serve as a temporal store for memories, while remote memory formation requires the reorganization of such stored information at the cortical level. Reactivation (or replay) of patterns of previous waking activities during “offline” periods was later suggested as a mechanism for such reorganization (148, 149). Sleep provides ideal slots for such offline consolidation as no incoming input is present, which allows the system to shift from “encoding” mode to “consolidation” mode. Such reorganization would also enable the mnesic trace to become gradually independent of the hippocampus, despite the fact that the initial encoding is dependent on both distributed cortical modules and the integrative function of the hippocampus which links the distributed aspects of the trace together. The idea of such a temporal gradient in the hippocampus-dependence of memories has been backed by many studies on anterograde amnesia which describe a temporal gradient for hippocampal-dependence of more or less remote mnesic traces. However, according to the description by Frankland and Bontempi (150), the episodic component of mnesic traces seems to escape such dissociation. This led to the multiple trace theory, according to which contextual and spatial information of a given mnesic trace remain dependent on the hippocampus despite the fact that the semantic component transfers to “cortical only” storage (151). Once again, this is coherent with AD, where episodic memory decline and a loss of hippocampal integrity are amongst the first signs of the disease. These observations converged giving rise to the currently accepted *active system consolidation* theory (152, 153). This framework is still based on the theory of systemic consolidation allowing remote semantic memories to claim their independence from the hippocampus and stipulates that this takes place with the help of repeated reactivation or replay of time-compressed activation patterns of previous wakeful periods (154).

Replay is thought to occur during NREM sleep and, to a lesser extent, during different wakeful behaviors (155). During these states, the synchronized activity of several oscillators enables the transmission of these traces from the hippocampus to the cortical networks. While this allows for stabilization and transfer of the mnesic traces to cortico-cortical centers during NREM sleep (systemic consolidation), REM sleep might be implicated in more local stabilizing processes (synaptic plasticity) (152, 156, 157). This role of REM sleep is also consistent with previous hypotheses which suggest a “local synaptic organizer” or “integrator” function for this specific sleep stage (158–160).

**Figure 3:**
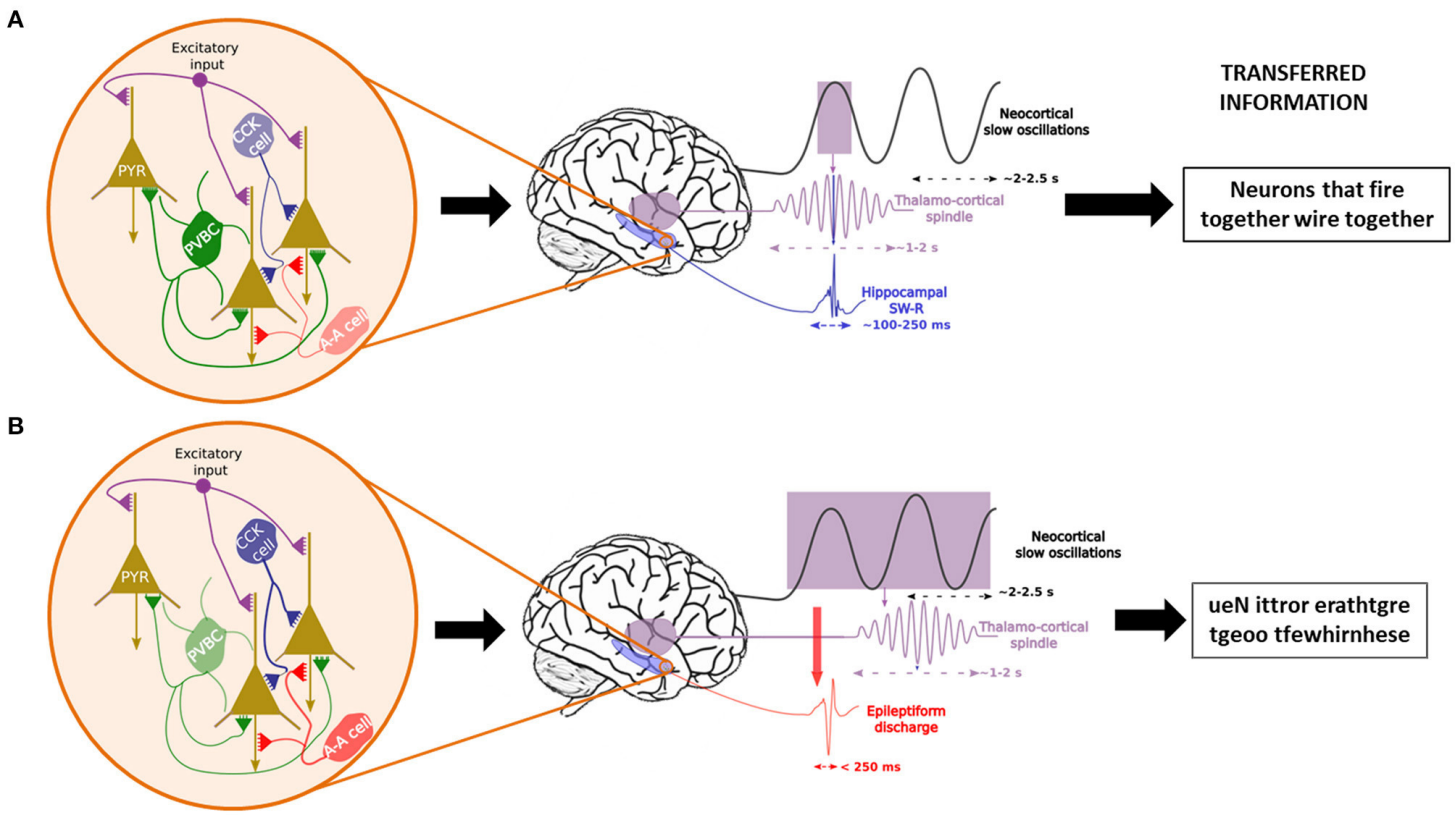
Temporal control of sleep-related oscillations and their disruption in case of interneuronal dysfunction. In physiological conditions **(A)**, Parvalbumin-positive basket cells (PVCB) are the main regulator of the firing patterns of pyramidal cells (PYR) in the hippocampus, leading to the generation and control of sharp-wave ripples (SW-R) nested in the trough of sleep spindles, themselves time-locked to the ascending phase of neocortical slow oscillations. This enables the transfer of correct information packets to the “long-term memory stores” from the temporary hippocampal reserve. In case of PVBC dysfunction **(B)**, other interneurons (CCK cells = Cholecystokinin interneurons, A-A cells = Axono-axonic cells) try to compensate for the diminished inhibitory tone. Nonetheless, they are insufficient due to their more distal synapses on PYR cells. This leads to uncontrolled excitation and epileptic events that may induce spindles (161) which leads to the consolidation of nonsense information.

As for the role of NREM sleep-related consolidation, three hierarchically organized, nested oscillatory patterns seem to be essential (Figure 3) (152, 162): cortical slow oscillations, thalamo-cortical spindles and hippocampal sharp-wave ripples. But what makes these oscillations so crucial for memory consolidation?

**Cortical slow oscillations (SO)** are ~0.75 Hz oscillations whose function in memory consolidation is suggested by the observed positive correlation between learning and the SO amplitude during the ensuing rest period (163). Moreover, inducing SOs via transcranial magnetic stimulation increases the consolidation of hippocampus-dependent memories (163, 164). The other role of these large-amplitude waves is the synchronization of neuronal activity by the creation of fluctuation between hyperpolarizing “down-states,” characterized by attenuated neuronal firing, and depolarizing “up-states” during which firing activity reaches levels observed during wakefulness (157, 165). Most notably, SOs also coordinate the occurrence of thalamo-cortical spindles and sharp-wave ripples (166) whose role in memory consolidation seems to be vital. However, in AD, the quantity of SOs decreases in an Aβ_1−42_ load-dependent manner (73).

The above-mentioned **thalamo-cortical spindles** are waxing-and-waning oscillations of 0.5–3 s between ~10–16 Hz. Spindles occur on the depolarized up-state of SOs (167), although the literature usually differentiates further between centroparietal fast (13–15 Hz) spindles, indeed phase-locked to the ascending phase of SOs and frontal slow (10–12 Hz) spindles that occur on later (~200 ms) phases of SOs reviewed by (168, 169). The timing of SO-spindle coupling have been reported to be related to hippocampus-dependent memory processes (170), while the density and duration of spindles during post-learning sleep has been continuously shown to be positively correlated with better performances on declarative or episodic memory-related tasks (171–174). However, during aging, their density and duration decrease, their frequency slightly increases (175–177), and their precise phase-locking on SOs is disturbed (178). These changes are even more marked in AD and MCI (179), which seems to be especially true for fast spindles (180), potentially further impacting memory consolidation efficiency (181, 182). Spindles are hypothesized to be essential to consolidation as they provide the network with a time window without interference from external stimuli (183, 184) during which information can travel from the hippocampus to the cortex.

The transfer of information itself relies on **sharp-wave ripples (SW-Rs)** that are physiological transients of 50–150 ms, with the ripple component in the 140–200 Hz frequency range (185). They arise from the hippocampus, with the sharp-wave component originating from the CA3 and the ripple being generated at the level of the pyramidal cells of the CA1 (185). Recent results also point toward the involvement of the CA2 sub-region as an initiator of the SW-R complex and as a potential origin of SW-Rs arising following learning tasks involving social memory (186, 187). Place-cell recordings in rodents (188) showed that time-compressed replay of past waking behaviors occurs during SW-R bursts that are nested in the troughs of spindles (189–191). SW-Rs are therefore regarded as the ideal “packages” of information transfer between the hippocampus and the neocortex (149, 166, 190, 192). Their role in memory consolidation has been suggested by experiments whereby ripple activity is selectively suppressed during post-training rest periods in rodents (193, 194) which led to poor post-rest recall performances. Moreover, as is the case with spindles, a marked increase in ripple activity was observed following learning in both rodents and humans. As we will see in the next paragraph, they are also altered throughout AD progression.

### Derailed Oscillations During Sleep and Epileptic Activity

The finely tuned oscillatory activity between SOs, spindles and SW-Rs can be seen as the meticulously synchronized playing of the many members of an orchestra. However, as with complex symphonies, at such high levels of precision there is always an increased probability for errors that can quickly derail into cacophony. This same risk is highly inherent in consolidation-related oscillations where a minor discrepancy suffices to push the system into a pathological state. In fact, even before the theories of memory consolidation, it was suggested that the mechanisms of remote memory formation and those of epileptic activities share many similarities (195, 196). Based on the well-known Hebbian principle (neurons that “fire together, wire together”), this early suggestion evoked a “hijacking” of learning mechanisms, be it learning or epileptic discharges. However, more recent results incited Halász and colleagues (197) to suggest that epilepsy (even simple EAs) is a derailment of plasticity-related mechanisms rather than an external force that sometimes profit from a pre-existing mechanism to wreak havoc in the brain. This risk is all the more increased for SW-Rs which are the most synchronous physiological oscillations that occur in all mammalian species (155, 185), as ~10–18% of hippocampal neurons, as well as neurons of the surrounding regions fire during SW-Rs (145). SW-R initiation is thought to be a result of the coincidental firing of pyramidal neurons, which generates a massive increase in the activity of the recurrent CA3 network, which leads to the SW-R [see (198) for more detail]. However, instead of unsupervised excitation, this activity is regulated by synchronized perisomatic inhibition by PVBCs which would be essential for maintaining the behavior of physiological SW-Rs [see (196, 199)]. However, when EAs are present, for example due to a dysfunctional transmission from PVBCs to pyramidal cells, there is a marked decrease in SW-Rs (161, 200). Remarkably, during such EAs, all other interneuron types step up their firing rates in response to the fallout of the perisomatic inhibition by PVBCs. Nevertheless, their influence on pyramidal neurons via dendritic synapses is not sufficient to suppress the induction of EAs (Figure 3) (196). What these results suggest altogether is that when PVBCs can respond to the synchronized excitatory activity of pyramidal cells, physiological transients (SW-Rs) are present, but in the absence of sufficient perisomatic inhibition, the result is a pathological SW-R, most often in the form of EA. This under-inhibition might be achieved either by dysfunctional PVBCs or by an abnormally rapid recruiting of pyramidal cells due to neuronal hyperexcitability with which PVBCs cannot keep up. As we have seen, both of these conditions are present in the AD brain and could explain the sleep-related nature of epileptic abnormalities. These results seem to converge harmoniously with the recent results provided by Caccavano et al. (201), who demonstrated in the 5xFAD transgenic mouse model of FAD that PVBCs receive unusually low levels of excitatory input during SW-Rs, which results in a reduction of almost 50% in their firing rate during SW-Rs. This hypoactivity of the PVBCs leaves the pyramidal cells with an enhanced excitation/inhibition ratio that is highly prone to EA. Even in *in vitro* mouse brain slices that only partially reproduce the network interactions, this gives rise to abnormal SW-Rs with decreased length, increased frequency and wider amplitudes. Interestingly, Jones et al. (202) described a predictor function of early SW-R activity decrease on subsequent spatial memory deficits in a knock-in rodent model (apoE4-KI) of AD. At the same time, two other teams observed decreased SW-R frequency and SO-SWR and SO-spindle coupling in two transgenic models of AD [(203) for 3xTg-AD and (204) for TgF344-AD].

Based on these findings, decreased SW-Rs would lead to less efficient memory consolidation. However, EA-transformed SW-Rs can inflict even more harm than detailed above. Notably, in a rat model of TLE, Gelinas et al. (161) provided further support for the SW-R-to-EA transformation and showed that EAs are associated with memory consolidation deficits. Moreover, they also found that EAs can lead to abnormal induction of spindles, especially during wakefulness but also during REM sleep, even though spindles never occur during this state in physiological conditions. Given that spindles are the safest highway of communication between the hippocampus and the neocortex, information could travel even for the abnormally induced spindles. However, as suggested by Buzsáki (185), these spindles might not carry learning-related traces which would lead to the consolidation of potentially useless or even scribbled cacophony arising from the EAs that elicited them (Figure 3). This conclusion is supported by Bower and colleagues (205), who, in an intracranial EEG (iEEG) experiment on mTLE patients, showed that neuronal assemblies activated during a seizure benefit from the most remarkable learning-associated plastic changes during the post-ictal sleep period. This suggests a potential detouring of sleep-related memory consolidation mechanisms by EA. Picking up on this thread, another team using iEEG recordings also described dysfunctions in memory consolidation stemming from sleep-related hippocampal ictal or interictal epileptic activities in epileptic patients (206). They recently completed this work by suggesting a model that explains the accelerated long-term forgetting often experienced by patients with epileptic activities during sleep (207).

## Ancillary Damage of Sleep-Related Epileptic Activity in AD

**Figure 4:**
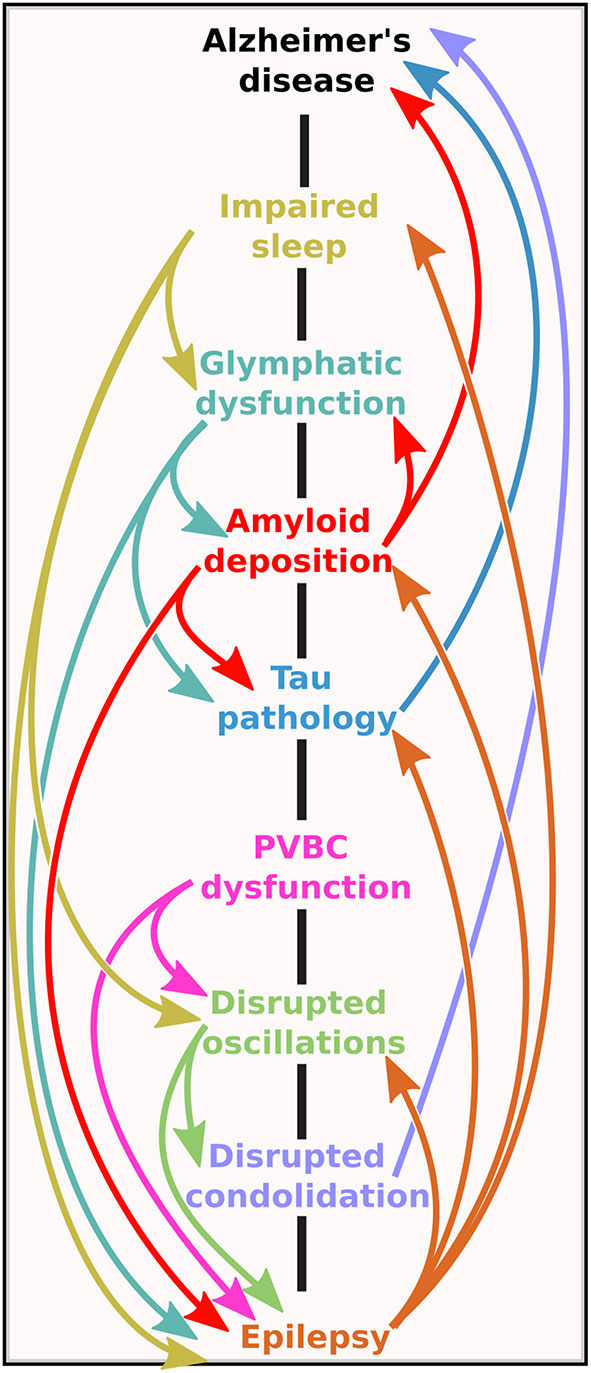
The bidirectional road between Alzheimer's disease and epilepsy. Note that the mechanistic pathways contain several vicious cycles, meaning that no matter at which level the initial imbalance is on the road, the self-amplifying potential of the system could lead to the onset of one (or possibly both) pathologies.

But sleep-related EA may not only inflict harm on the brain by derailed oscillatory patterns but also by disrupting sleep itself which fulfills many other essential functions besides memory consolidation. We have already noted that EAs are predominant during REMS in rodent models of AD. Therefore, EAs, or their induced spindles, which interrupt REMS, could increase network hyperexcitability by disrupting downscaling functions generally attributed to REMS. In fact, although the role of REMS in memory consolidation is still disputed, findings point to a role in local, synaptic plasticity-related processes, including an impact on the expression of immediate early genes related to consolidation (*ASH framework)*, and a general downscaling of synapses leading to the elimination of weak synapses (*Synaptic Homeostasis Hypothesis)*. Downscaling of synapses is essential since the sum of synaptic weights in the brain should maintain a quasi-constant value to avoid hyperexcitability (145). The probability that REMS is implicated in such processes is all the more increased since evidence shows that firing rates decrease brain-wide after the short periods of REMS (159). Therefore, the interruption of theta oscillations during REMS by EAs or induced spindles might lead to an incomplete reduction of synaptic weights and elevated baseline neuronal activity, further aggravating pre-existing activity. This self-aggravating cycle between sleep and epilepsy could add yet another vicious cycle to the path between epilepsy and AD (Figure 4). Finally, as we mentioned earlier (see paragraph 1.4), we should not forget that even without EA, sleep structure in itself is already impaired early in the course of AD (208), which might further aggravate both memory consolidation and the epileptic phenotype.

We have seen the various pathways through which epileptic activity might aggravate the progression of AD (summarized in Figure 4). The concerning number of vicious cycles in this system seems to justify considering an amelioration of epilepsy-detection in AD patients and the treatment options of these aberrant network activities.

## Methodological Considerations, Diagnostic Hurdles and Treatment Options

### Methodological Considerations for Research

As it has been emphasized throughout this review, the prevalence of EA in AD is highly variable across studies, which we suspect is the result of methodological choices. In fact, several points of divergence create a snowball effect of inconsistencies in the results.

One such point is the diagnostic criteria of Alzheimer's disease. While most papers follow NINCDS-ADRDA or IWG-2 criteria, some authors base the diagnosis on MRI markers (26), or do not differentiate between AD and other types of dementia (116, 118, 129, 209, 210). Finally, retrospective or prospective population-based studies or projects using datasets from large memory clinics or databases cannot verify the diagnostic method for all patients in the cohort (122, 123, 127, 128, 131, 132, 211). Using only imagery and neuropsychological data for diagnosis might inflate the percentage of “AD” patients with epilepsy, given that patterns of cortical atrophy and cognitive symptoms can be highly overlapping in epilepsy and AD (212) or aMCI from which the conversion rate to AD is exceptionally high (110). On the other hand, while approximately two-thirds of all dementias are due to AD, mixed cohorts might also impact the observed prevalence of epilepsy and dementia due to AD.

Another important consideration is study type. Many previous publications consisted of retrospective cohort studies, which, besides having an inherent risk of including a relatively high percentage of misdiagnosed dementia patients, are often carried out by a meticulous sweep of patients' medical history [e.g., (123)]. Even when the problem of misdiagnosis is averted by the availability of post-mortem pathology reports confirming AD [as in (126)], using medical reports to infer the presence of epilepsy brings in several biasing factors. First of all, although convulsive seizures might be observed by caregivers, they might not be reported by the patients themselves, who might not recall such events. Secondly, while in some cases EEG results are available in the medical history, they are primarily standard 20–30-min EEGs without periods of sleep (25, 26). In fact, we have discussed at large that subclinical network anomalies or silent seizures in AD occur mainly during sleep (24–26). Accordingly, Horvath et al. (213) have shown that a 1-h EEG recorded between 0:00 and 8:00 (potentially including periods of sleep) is 16.5 times more sensitive in detecting epileptiform events than a recording of the same length during the day (from 8:00 to 16:00, mainly containing wakeful sections). Moreover, recording length strongly correlates with EEG sensitivity. Therefore, there is no comparability between results obtained with 30-min recordings and those obtained after eight or even 24-h recordings.

Another critical point is the question of inclusion and exclusion criteria. While it is now well established that EA can occur at any stage of the disease, even before symptom onset (25, 112), its prevalence still shows strong correlations with disease severity. Therefore, the form of AD or the inclusion threshold for disease severity (measured by MMSE, CDR, ACE or any other commonly used neuropsychological evaluation) could also influence results. This also applies when deciding whether or not to include patients with a known history of epilepsy (or even with ASM treatment). In such cases, separating the results according to pre-existing epilepsy, as done in the recent article by Lam et al. (24), could be a solution to avoid bias. However, other neurological antecedents (e.g., stroke, tumors, previous head trauma, etc.) and pharmaceutical treatments that suggest underlying psychiatric or neurologic comorbidities (such as antipsychotics, antihistamines, benzodiazepines, and of course, ASMs) should be considered in exclusion criteria (24). Moreover, a large proportion of AD patients are prescribed antidepressant medication, even in the early stages, and including these patients presents another issue. While such medication might not have a substantial effect on seizure susceptibility (214), it strongly impacts the sleep cycle, especially REM sleep and sleep fragmentation (215). As mentioned earlier, EA in AD is strongly linked to sleep. Therefore, including patients who are on medications that significantly modify the sleep-wake cycle has a high probability of interfering with EA detection.

Another critical question is whether, technically speaking, EA in AD can be diagnosed. First of all, for obvious medical and ethical reasons, EEG is recorded using scalp electrodes in the absence of drug-resistant epilepsy. However, as it was demonstrated in a study using implanted Foramen Ovale (FO) electrodes in two AD patients, ~95% of the spikes captured by iEEG are not detected on simultaneous scalp EEG recordings (135). Furthermore, this finding was also echoed by a case-study using FO electrodes in a fronto-temporal dementia patient, indicative of the epileptic phenotype being a shared feature of several neurodegenerative diseases (216). Moreover, even with surface electrodes, most of the EA are captured at the level of the temporal lobe (24, 25, 135). However, they are often only detected by temporo-basal electrodes, which are not included in the electrode set used in most setups. Finally, other methods such as MEG have the potential to rank between iEEG and surface electrodes, but MEG has only been applied in two studies on this topic so far with quiet discordant results (25, 113), and given the scarcity of studies having access to MEGs, this incoherence is not likely to be remediated in the near future. These issues already present a bias toward high underestimation of EA in AD. However, the more pressing problems are the detection method and sensitivity. In fact, the majority of authors used manual detection by experts, with some papers relying on one expert (111), or two neurophysiologists (26) or epileptologists (119) or a combination of these specializations (25). Most, but not all experts are blinded to the condition of the participants (AD or control) while analyzing the data, which could introduce further bias. Another problem that probably prevents EA detection, even for automatic detection, is the modified characteristics of brain activity in AD patients. In fact, their EEGs show more significant slowing than is seen during healthy aging (141, 142), which is most prominent during REM sleep (217). Therefore, these EEGs show a significant amount of slowing-related activities, which makes the classification of some pathological or unusual but physiological activities rather tricky.

Finally, other issues regarding the scoring of EEG activity are epileptic transients of sleep or sporadic sleep spikes (SSS) and temporal intermittent rhythmic delta activity (TIRDA) which have already been linked to mTLE, but which have not yet been established as pathological events. However, as Lam et al. (24) pointed out, a surprising increase in SSS is seen in AD patients with underlying EA. Defining when these events should be re-evaluated would be of interest to improve the characterization of EA as opposed to benign EEG patterns. It is all the more important since the boundary where EEG patterns are considered as subclinical or interictal activities, is already blurred. The most common definition that authors give for spikes is “sharp waveforms 20 to 200 ms, clearly distinct from ongoing background activity, with an associated subsequent slow-wave” (25, 26), but this is not unified across studies. In fact, the prevalence dropped from 42 to 48% respectively in the Vossel and Horváth studies to as little as ~6% in the study by Brunetti et al. (113), who defined EA based on stricter morphologic characteristics. However, contrary to preclinical studies (218, 219), there are no recommendations or guidelines for EA detection in AD patients, considering the many abnormalities and specificities already present on EEGs. Such recommendations would greatly facilitate the diagnosis of epilepsy or subclinical EA in AD patients. In their article, Sen and colleagues (220) have already made a step toward formulating such guidelines for neuropsychological data collection. They suggest standardized, culture-independent, short batteries of tests, with baseline evaluation and longitudinal tracking. Based on snippets from existing studies and an ongoing clinical study (ClinicalTrials identification: NCT03923569), we suggest some methodology-unifying considerations concerning the measurement and detection of epileptic events in AD in Textbox 1 in the hope of amplifying data on mechanisms that have already been suggested but which have still not been replicated.

**Textbox 1:**
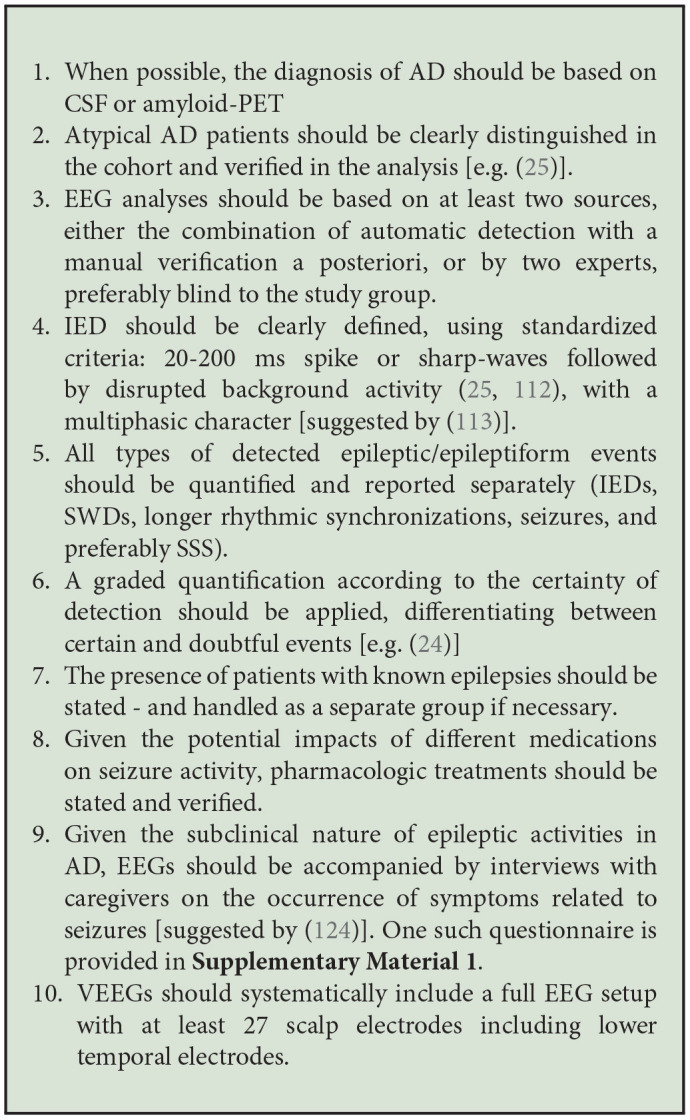
Methodological considerations for clinical research.

### Diagnostic Hurdles in Clinical Practice

As is the case of all AD-related issues, AD-related neuronal hyperactivity should be treated as soon as possible, especially since AD patients with EA seem to benefit from such treatment, as we will see below. However, the detection of subclinical epileptic activity is subject to high methodological variations, not only in research but also in clinical practice. This variability in methodology might be one of the reasons why this issue is not yet profoundly nested in current clinical practice. As we have emphasized throughout this review, EA is mainly subclinical in AD (221) and often goes unnoticed by the patient and the caregiver(s). Hence, even if periods of loss of contact or stereotypical behaviors/automatisms are present during ictal or interictal activities, they might be considered part of the disease progression rather than an epilepsy-related event. To address this issue, questionnaires or interviews targeting these symptoms could be applied preferentially in the presence of caregivers [an example of such a questionnaire, elaborated from previous observations (26, 221–223) is provided in Supplementary Material 1].

In any event, unless overt signs of epilepsy are present, EEG, especially of longer duration including periods of sleep, is rarely requested for AD patients. An exception applies to patients with suspected sleep apnea who undergo a polysomnographic examination coupled with EEG. In fact, until portable, easily applicable, useable-at-home technologies that measure brain activity during the night become widely available, more systematic screening for epileptiform activities in AD is unlikely to take place. Although FO electrodes are especially well-positioned to capture EA from anterior temporal and mesial-temporal structures, they are not feasible for the entire AD population due to the invasive nature of the procedure. Video-EEG also seems to be a relatively reliable method. However, most healthcare systems across the globe are probably unprepared to screen the entire AD population. The study by Vossel (25) indicated that MEG might be a highly sensitive non-invasive detector of EA, especially if it can be backed by computational approaches that enable the detection of signatures of EA even in the absence of large amplitude deflections (33, 224). However, MEG also requires expensive, specific and, more often than not, inaccessible equipment for EA screening.

On the plus side, although methodological differences led to highly variable incidence rates across previous studies (see Table 1), the different inclusion criteria helped to narrow down the patient group that is at higher risk for AD and EA comorbidity and for whom a vEEG screening would be the most important. For sporadic cases, this includes patients with an earlier age of onset (<65 years), APOE4 allele(s), higher educational levels or a very apparent discrepancy between cognitive symptoms and the degree of brain atrophy during the early stages (indicative of a high cognitive reserve). In FAD, given the extremely high prevalence of epilepsy (11) and the relatively low number of patients affected by this condition (1% of all AD cases), systematic screening by v-EEG at cognitive decline onset should be conducted. Detecting and treating EA before seizures with high levels of network reorganization potential might help to slow down cognitive decline.

**Figure 5:**
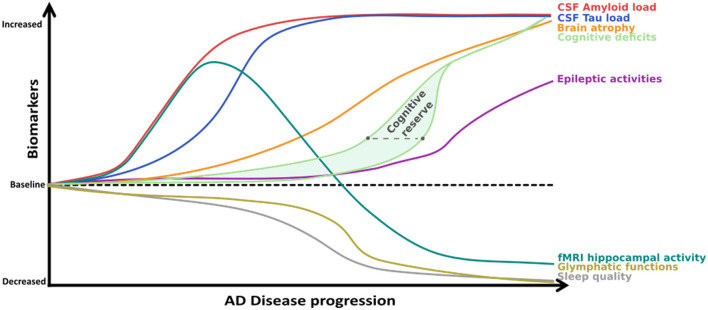
Proposed model of biomarker-and cognitive decline progression over the course of AD. High cognitive reserves compensate on a functional level for marked Amyloid and Tau aggregation and atrophy may lead to delayed diagnosis. This might explain why EA is most often detected in highly educated participants who are (seemingly) in the mild stages of the disease. However, once compensation is no longer possible and EA becomes more frequent or probable, the cognitive decline might be steeper, which is compatible with the fast progression noted in AD patients with E/EA. Figure adapted from (33) with permission. The gray dashed line represents the approximate time of diagnosis.

Another crucial diagnostic consideration is that the link between AD and epilepsy is increasingly considered to be a bidirectional road (106, 225). It has been shown that while AD patients have an increased risk of developing epilepsy, epilepsy patients also present a 2-3.6-fold risk of developing dementia compared to the non-epileptic population (106, 129). Unfortunately, in current clinical practice, just as AD-related silent EA often goes unnoticed, there is also a risk that instead of being diagnosed as AD, the cognitive complaints of epilepsy patients might be mislabeled as Epileptic Amnestic Syndrome, a less severe condition with good responsiveness to ASMs (221, 226). This could lead to suboptimal treatment plans for dementia. But even if a diagnosis of AD is not justified, an altered path of cognitive decline in epileptic patients compared to healthy older adults has been observed throughout the literature, as was summarized by Breuer and colleagues (2016) (227) in favor of *accelerated cognitive aging* for people living with epilepsy. According to their review, while a younger age of epilepsy onset seems to be a risk factor for such an increase in cognitive decline, it was emphasized that LOEU patients are no exception. As they underscored, the brain of older adults tackles several problems at a time, such as elevated levels of inflammatory brain response, comorbidities that affect cognition (such as AD), the use of several medications with often interacting effects, and, notably, a potentially decreased cognitive reserve. This decrease means that they cannot compensate for epilepsy-induced cognitive problems like younger epileptic patients, which leads to an even more accelerated cognitive decline (227). This is consistent with the finding that AD patients with epilepsy generally have higher educational levels (25, 26). In fact, patients with high cognitive reserves can compensate even for comorbid AD and (subclinical) epilepsy for longer periods, and therefore present only mild symptoms even in more advanced stages of AD in terms of biomarkers. However, once the macro- and microstructural damages reach a level where compensation is no longer possible, the disease seems to progress at an excessive speed, as is the case for AD patients with EA (Figure 5) (25, 130, 137). However, this conclusion might beg the question of what happens to AD patients with EA with more modest cognitive reserve. It might be that the accelerating effect of the comorbid pathologies without the shield of cognitive reserve is such that polysomnographic examination might not be possible in some cases even shortly after diagnosis due to agitation.

To conclude on the bidirectionality of the link between AD and epilepsy, we would like to address a notion from recent work by Sen and colleagues (220). They noted that it could not be ascertained whether epilepsy simply facilitates dementia by lowering brain reserves or whether it produces it. According to our interpretation, both suggestions are valid: (sub)clinical epilepsy, by continuous insults to neuronal circuits and through all the harmful effects we reviewed, leads to an increase in “AD-inducing” substances (especially if clearance mechanisms are impaired) and, through the reorganization of neuronal networks, reduces brain reserve that compensates for dementia-related cognitive loss. In any event, screening of epilepsy patients for AD biomarkers, especially those at higher risk for dementia, should be systematic to enable earlier detection which might ensure a more efficient, partially preventive (228), partially medical treatment. This applies to patients at risk for AD, including those with LOEU, patients with cognitive complaints, or a family history of dementia. The importance of this screening came to light through a recent publication (106) which found abnormally decreased CSF Aβ1-42 levels in LOEU patients, with a hazard ratio of 3.4 for progressing to AD. That is why the medical recommendations that summarize the most important notions for clinical practice from this paragraph (Textbox 2) include both pathologies. This seems all the more justified since epileptic auras have also been described as preceding MCI onset by 4–7 years, leading to the suggestion of an “epileptic variant” of AD (222). Of note, cognitive functions (measured by MMSE scores) remained relatively stable at the one-year follow-up for almost all participants, suggesting a potentially positive impact of ASMs in preventing a disease course that is noted to be generally worse for the AD-epilepsy comorbidity (222).

**Textbox 2:**
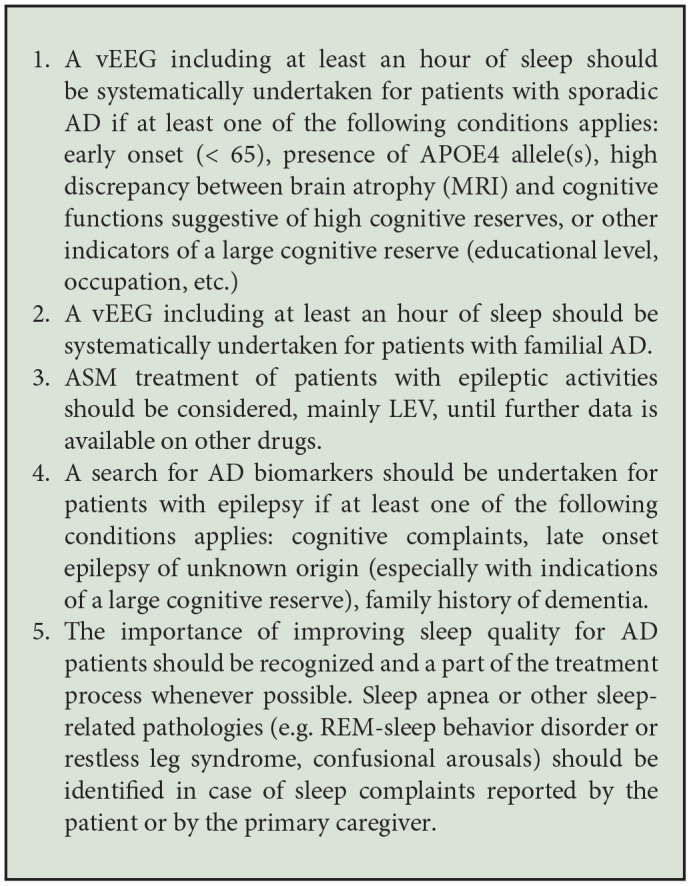
Recommendations for clinical practice.

### Treatment Options and Considerations

Despite the widespread harmful effects of EA, it is quite probable that its attenuation in AD patients is not the star that sashays on the red carpet and dazzles the world with an all-in-one cure to AD. However, silencing network hyperactivity might lead to slower disease progression, a decrease in disease burden and should be considered as part of the standard medical treatment plan for AD patients. In 2007, it was estimated that delaying AD onset by as little as a year could lead to 9.2 million fewer cases in 2050 (229). A one-year delay could have a substantial impact on people living with AD and could also have major socioeconomic impacts, given that the global cost of dementia was estimated at US $818 billion per year in 2015 (230), most of which can be attributed to AD.

We have just explored whether neuronal hyperactivity might accelerate disease onset and progression. If this is the case, treatment in AD patients at the earliest possible moment might delay disease progression. A rather impressive quantity of literature is now available on treatment options for AD-related epilepsy and was recently reviewed at length by two separate research groups (33, 231). These results accentuate the positive effects of Levetiracetam (LEV) both in rodent models and in AD patients. In fact, LEV led to improved memory performance in aged rats in the study by Koh et al. (232). Interestingly, the treatment only benefited older but not younger animals, suggesting that this ASM treats excess neuronal activity without inducing potentially detrimental decreases in baseline activity. This preclinical result was also replicated in a recent clinical study with AD patients in mild-moderate stages of the disease (MMSE > 18) in which a marked improvement in executive functions (measured by the Stroop test) and in navigation capacities (as shown by results on a virtual route learning test) were noted after only 4 weeks of treatment with low doses of LEV (125 mg/day) (114). Importantly, beneficial effects in the above-mentioned tests were only observed for patients with EA, further suggesting that LEV targets only excess neuronal activity and that this excess activity impairs cognitive functions. Among the other clinical studies on ASM treatment in AD-related EA, that of Cumbo and Ligori (233) should be noted, where the impact of long-term use of several ASMs (LEV, phenobarbital (PB) and lamotrigine (LTG) in AD patients with concomitant epilepsy was examined (n = 38, 28 and 29 respectively in the three treatment arms). They found similar responder rates between the three ASMs (71% responded to LEV, 59% to LTG and 64% to PB) which were also comparable to the rate noted in epilepsy unrelated to AD (~66%). However, while LEV had relatively few side effects and slightly improved cognitive functions, PB negatively impacted cognitive functions. On the other hand, LTG was associated with a slight decline in cognitive functions but with an improvement in mood. Given the encouraging results with LEV, it is not surprising that most clinical trials tested this medication rather than other ASMs. For example, another study of chronic treatment with LEV in cases of severe AD with late-onset seizure found that 72% of the patients were seizure-free after the one-year follow-up, only 8% were non-responsive, while 16% had undesirable side effects (234). In addition, in a study with a small sample size of 7 AD patients, Musaeus et al. (235) described normalized EEG activity after a single dose of 7.5 mg/kg (~500 mg) of LEV but not after 2.5 mg/kg. This normalization was manifested by decreased coherence in slower and increased coherence in faster bands. In two other studies with aMCI patients, Bakker et al. (236, 237) showed that a 2-week treatment with lower doses of LEV (most notably 125 mg) lead to decreased hippocampal hyperactivity (as measured by fMRI signals in the CA3 and the entorhinal cortex). Although this relatively short-term treatment did not seem to affect long-term memory performances, it did improve pattern separation, which is an important component of episodic memory. Furthermore, it appears that ASMs (including LEV) do elicit such a positive effect on cognition even with chronic treatment in LOEU patients without AD (238), especially in patients diagnosed with focal seizures. Although the amount of randomized case-control studies remains too low for further conclusions (231), preclinical results of antiepileptic treatments in AD are promising [reviewed by (27), and (239)]. They suggest decreases in excess neuronal activity (61, 240), improved cognitive functions (240–242), reduced amyloid plaque load in some cases (242), or a rescue from the depletion of neuronal stem cells due to network hyperactivity [(241), see (27) for a review]. LEV also seems to prevent tau-dependent depletion of the potassium channels of the Kv4.2 type which were mentioned earlier as being implicated in neuronal hyperexcitability and further aggravate the above-mentioned vicious cycles (61). Finally, LEV restores impaired LTP function in the perforant path toward dentate granule cell synapses and normalizes levels of NPY, Fos and calbindin proteins, all of which are related to excess neuronal activity (240). Moreover, many potential pathways for treatment are under investigation, and have been reviewed recently (33).

It is important to note that the data that is currently available mainly describes the effect of LEV although other, less explored molecules should also be tested, especially given the circadian variations of EA activity in AD patients. In fact, since EA are predominant during sleep, a posology including a bedtime dose of ASM might be the most effective way to tackle these anomalies. Moreover, limiting the strongest effect of the ASMs to sleep intervals could also prevent most of their side effects during wakefulness. On the other hand, the half-life of LEV is rather short, and even residual impacts are improbable after an entire night. Consequently, taking it before sleep could lead to an overspill of EA during wakefulness. This might be a rationale for testing other available treatments with a longer half-life. However, until more data are available on the subject, LEV seems to be the most effective treatment of EA for AD patients of all the rarely tested ASMs in this field.

Finally, sleep-related EA might only be one of the ways through which sleep (and related consolidation processes) is altered in AD. We have seen that fragmented sleep could affect amyloid and Tau clearance from the brain by impeding glymphatic functions, and it has been shown that altered sleep quality is linked to Tau and Amyloid deposition alike (76, 243). Attempts at remediating fragmented sleep (due to apnea, confusional arousals, restless leg syndrome, REM sleep behavior disorder or other causes) should be made as early as complaints concerning sleep quality are reported by the patient or by their caregiver(s), especially since the literature on the close relationship between sleep quality and cognitive decline is growing daily. A morning dose of donepezil has been suggested to counteract REM sleep decline and REM-sleep related EEG slowing. However, other pharmacological treatments are still under investigation for AD (208). Non-pharmaceutical treatments such as bright-light therapy show encouraging results and could be considered, along with psycho-educational measures and behavioral changes aimed at improving sleep (daily physical activity, less time spent in bed during the day, more time spent outside in natural light) (208).

## Conclusions

In this paper, we reviewed how the often behaviorally masked epileptic phenotypes in AD (or possibly, vice versa) might be unmasked and aggravated during sleep. Sleep is a crossroads where AD-related proteins can propagate unsupervised and when epileptic activities are more likely to happen through the occurrence of highly synchronized consolidation-related neuronal activities in under-inhibited networks. We also aimed to draw attention to the risk that such activities might cause cognitive decline, in particular, difficulties with memory consolidation and further insult to the already fragile hippocampal formation by chronic reorganization of neuronal circuits. We emphasized the importance, or rather, the absolute necessity of early screening during medical care, for both epileptic events in AD and AD biomarkers in epilepsy, to enable early medical intervention and to prevent the escalation of neuronal and generalized brain damage. Given that such screening is impossible in the entire AD and epileptic population, we filtered homogenous results from previous findings to characterize patients at risk. Finally, we briefly summarized the available knowledge on the relationship between AD and epilepsy, which has grown incredibly since the first observations of Blocq and Marinesco (3) and is still growing daily. However, it will be challenging to arrive at clinically relevant conclusions from coherent results without unified methodologies across research teams. As others have noted before us, such coherence will only be possible by comparing cross-study samples, or through joint data acquisition in large-scale collaborations.

## Author Contributions

ABS contributed to the conceptualization of the review, collected references, and drafted the manuscript. BC, LD, EB, JC, JP, and LV contributed to the conceptualization of the review and provided feedback. JC and BC organized references. FG and JC devised the supplementary questionnaire and provided feedback. LV and LD supervised the project. All authors contributed to the article and approved the submitted version.

## Funding

This research was supported by the French organizations Fondation Alzheimer (Project EREMAD), France Alzheimer and the Agence Régionale de Santé (ARS).

## Conflict of Interest

The authors declare that the research was conducted in the absence of any commercial or financial relationships that could be construed as a potential conflict of interest.

## Publisher's Note

All claims expressed in this article are solely those of the authors and do not necessarily represent those of their affiliated organizations, or those of the publisher, the editors and the reviewers. Any product that may be evaluated in this article, or claim that may be made by its manufacturer, is not guaranteed or endorsed by the publisher.

## Abbreviations

Aβ: Amyloid-β peptide
ACE: Addenbrooke's Cognitive Examination
AD: Alzheimer's disease
APP: Amyloid Precursor Protein
AQP-4: Aquaporin-4
ASM: Antiseizure medication
(a)MCI: (amnestic) Mild Cognitive Impairment
BZD: Benzodiazepines
CDR-SB: Clinical Dementia Rating Scale–Sum of Boxes
CSF: Cerebrospinal Fluid
EOAD: Early-onset Alzheimer's disease
EA: Epileptic/Epileptiform activity
FAD: Familial Alzheimer's disease
IED: Interictal epileptic discharge
iEEG: intracranial EEG
LTG: Lamotrigine
LEV: Levetiracetam
LOAD: Late-onset Alzheimer's disease
LOEU: Late-onset epilepsy of unknown origin
MMSE: Mini-mental State Examination (Folstein Version)
(m)TLE: (Mesial) Temporal Lobe Epilepsy
NFT: Neurofibrillary tangle
PH: Phenytoin
(N)REMS: (Non)-Rapid-eye movement sleep
PSEN: Presenilin
PSG: Polysomnography
PVBC: Parvalbumin positive basket cells
SSS: sporadic sleep spikes
SWD: Sharp-wave discharge
SW-R: Sharp-wave ripple
SO: Slow oscillation
v-EEG: video-electroencephalography.
