# Supplementary material for: Sleep: The Tip of the Iceberg in the Bidirectional Link Between Alzheimer's Disease and Epilepsy
Source: Front Neurol. 2022 Apr 11;13:836292. doi: 10.3389/fneur.2022.836292 (PMC9035794; doi:10.3389/fneur.2022.836292)
Supplement: Supplementary file 1 [file Data_Sheet_1.PDF]

Supplementary Material 1 - Epilepsy questionnaire

Instructions: This questionnaire is intended to explore the occurrence of symptoms indicative of prior epileptic seizures. Given the frequent losses of consciousness or amnestic post-ictal episodes, it is preferred for the interview to be conducted in the presence of a caregiver or a person living with the patient.

Presence of caregiver: Yes ☐ No ☐

	ACCORDING TO THE PATIENT		ACCORDING TO CAREGIVER (IF APPLICABLE)	
	YES	NO	YES	NO
1- SENSORY DISTURBANCE (UNPROVOKED PAIN, TINGLING, PRICKING, NUMB SKIN)	☐	☐	☐	☐
2- UNEXPLAINED SENSATION OF FEAR	☐	☐	☐	☐
3- TRANSITORY MEMORY IMPAIRMENT	☐	☐	☐	☐
4- OLFACTORY HALLUCINATIONS	☐	☐	☐	☐
5- DEJA-VU	☐	☐	☐	☐
6- TRANSIENT MOTOR DISTURBANCE (LOSS OF MUSCLE TONE, PARESIS)	☐	☐	☐	☐
7- TRANSITORY LANGUAGE IMPAIRMENT	☐	☐	☐	☐
8- FEELING OF ASCENDING EPIGASTRIC WARMTH	☐	☐	☐	☐
9- OROFACIAL AUTOMATISMS	☐	☐	☐	☐
10- MOTOR AUTOMATISMS (E.G. RHYTMIC OR STEREOTYPED MOVEMENTS)	☐	☐	☐	☐
11- STRONG VERTIGO	☐	☐	☐	☐
12- AUDITORY HALLUCINATIONS	☐	☐	☐	☐
13- SUDDEN ONSET OF CONFUSION	☐	☐	☐	☐
14- ABNORMAL MOUVEMENTS (MUSCLE TWITCHES/MYOCLONUS)	☐	☐	☐	☐
15- LOSS OF CONSCIOUSNESS	☐	☐	☐	☐
16- CONVULSIVE SEIZURES	☐	☐	☐	☐
17- AUTONOMIC SENSATIONS (TACHYCARDY, TRANSPIRATION,	☐	☐	☐	☐
18- URINARY LEAKING AT AWAKENING	☐	☐	☐	☐
19- TONGUE BITING AT AWAKENING	☐	☐	☐	☐
20- TRANSITORY ALTERED STATE OF MIND, LAPSES IN AWARENESS	☐	☐	☐	☐
